# Hepatitis C Virus infection in apparentenly healthy individuals with family history of diabetes in Vom, Plateau State Nigeria

**DOI:** 10.1186/1743-422X-6-110

**Published:** 2009-07-20

**Authors:** Obinna O Nwankiti, James A Ndako, Georgebest ON Echeonwu, Atanda O Olabode, Chika I Nwosuh, Ema M Onovoh, Lilian A Okeke, Jumoke O Akinola, Boniface N Duru, Ijeoma O Nwagbo, Godwin O Agada, Anthony A Chukwuedo

**Affiliations:** 1 Viral Vaccines Production Division, National Veterinary Research Institute Vom, Nigeria; 2 Federal College of Veterinary and Medical Laboratory Technology, National Veterinary Research Institute Vom, Nigeria

## Abstract

Hepatitis C virus (HCV) infection is an important public health problem worldwide. Its association with, and predisposing nature for diabetes mellitus (DM) has been long established. This research was carried out to determine the prevalence of Hepatitis C virus (HCV) amongst people with possible genetic predisposition to diabetes mellitus living in and around Vom, Plateau State, Nigeria. 188 subjects were screened after they filled a structured questionnaire to determine some of their demographic data, social habits and possible risk factors. 5 ml of blood was collected from each subject and sera separated out. Biotech's third generation ELISA Kit for HCV antibodies was used for the screening. Liver enzyme analysis was carried out on positive samples to determine their disease status. A prevalence of 14.36% was recorded with the highest seropositive group being those in the age bracket of 18 – 37 years. 13(13.40%) of males and 14(15.38%) of females were sero-positive. Liver enzyme analysis of sero-positive subjects showed increased levels which may imply early onset of liver damage. These result showed that these individuals could later suffer diabetes which may be triggered by their HCV infection if not treated. This is not over-looking the economic significance of their ill health, assuming they progress to cirrhotic HCV or develop hepatocelluar carcinoma due to HCV chronicity.

## Background

Hepatitis C virus (HCV) infection is an important public health problem [1] affecting more than 170 million people worldwide [2]. HCV is a positive, single-stranded RNA virus in the Flaviviridae family. The natural course of Hepatitis C virus infection shows variability among individuals and depends on several factors. History of blood transfusion, tattooing, intravenous drug abuse, hemodialysis, abortion, nondisposable needle exposure, and frequent dental procedures are all common routes for contracting HCV infection [3]. In Nigeria, Halim and Ajayi reported that the prevalence varied between 5.8% – 12.3%. In another survey in Jos Nigeria, Onwuliri et al. [4] recorded a prevalence of 5.56%. Approximately 20–30% of infected persons clear the virus from their bodies during the acute phase. The remaining 70–80% of infected persons may develop chronic hepatitis which could progress to cirrhosis and hepatocellular carcinoma in 20–30 years [2].

HCV infection was found to be an independent risk factor associated with type-2 diabetes mellitus (DM) by multivariate analysis [5,6]. Age (> 57 years), family history of DM, body mass index (> 25 kg/m^2^), and previous interferon treatment are all independent factors for the development of type 2 DM in patients with chronic hepatitis C [7]. In 2000, Mehta et al. [8] reported a link between DM and HCV status in a representative sample of the general population of the USA. After adjustments for DM confounding factors such as age, race, high body mass index, and low socioeconomic status, they found that persons older than 40 years of age with HCV infection were 3 times more likely than those without HCV to have type 2 DM (odds ratio: 3.77; 95% confidence interval: 1.8–7.87).

Hepatitis C has clearly been demonstrated to be a precipitating factor for diabetes but only in patients with risk factors to develop such [8]. People with hepatitis C virus (HCV) infection appear to be at increased risk of developing type 2 diabetes. Patients infected with HCV are 3–5 times more likely to have type-2 DM than those without HCV [8,9]. A 2- to 10-fold increase in diabetic cases has been reported worldwide in HCV positive patients compared with liver disease control subjects [6,9-12]. In English type 2 diabetic subjects with abnormal serum aminotransferases, HCV antibody was detected in 28% of patients of African origin, 12% of Caucasians and 8% of Asians [13].

It is unclear as to why some patients with HCV infection develop diabetes because the pathogenic mechanisms leading to DM in patients with HCV infection are still not well understood. Both insulin resistance and impaired insulin secretion have been considered to play an important role in the development of DM. However, it is tempting to speculate that HCV infection is able to trigger autoimmune mechanism(s) against the insulin producing pancreatic beta cells in susceptible individuals. Genetic susceptibility for the development of DM type1 has been well documented in some individuals [14]. The major mechanism appears to be insulin resistance which is related to fibrosis score [15,16]. However, this cannot be the sole mechanism since the prevalence is also increased compared to other liver diseases. One possibility that was not highlighted is the association between hepatitis C and DM is iron overload which may partly explain already observed findings [17].

About 75 percent of patients with acute hepatitis C ultimately develop chronic infection. Researchers estimate that at least 20 percent of patients with chronic hepatitis C develop cirrhosis, a process that takes at least 10 to 20 years. Liver failure from chronic hepatitis C is one of the most common reasons for liver transplants in the United States. Knobler, *et al*. [17] reports an increase in DM type-2 before the development of advanced liver cirrhosis. Zein *et al*. found that, after excluding chronic hepatitis C patients who received previous interferon treatment, higher fibrotic stages in liver histology and family history of DM were closely associated with higher prevalence of DM and impaired fasting glucose in patients with chronic hepatitis C [18].

Hepatitis C virus and DM have been known to trigger each other but which disease predisposes more to the other is yet to be determined. Genetic predisposition to diabetes, though unmanifested, may be triggered by HCV. It only is necessary to determine the HCV status of those assumed to be genetically predisposed (i.e. family history of DM) so as to reduce/prevent their chances of manifesting DM.

Diabetes in this area in prevalent and most times is triggered or complicated by co-infection with other undiagnosed disease like HCV. This study is aimed at preventing such situations from arising in the subjects studied.

## Results

Out of the 188 subjects screened for anti HCV, 27(14.36%) were sero-positive while 161(85.64%) were sero-negative as shown in table 1. 97(51.60%) were males of which 13(13.40%) were seropositive. 91(48.40%) were females of which were 14(15.38%) seropositive, as shown in table 1. 10(5.32%) of those within the age range 18 – 27 years were seropositive. This age group gave the highest seropositive result as shown in Table 2 and 3. As cited in target subjects, all had history of Diabetes. Other demographic data in relation to screening results are as seen on tables 2, 3 and 4. Liver enzyme analysis on sero-positive subjects showed that 2(2.06%) men had AST levels above normal while 4(4.12%) men had ALT levels above normal. 3(3.29%) women had abnormal levels for AST and ALT as seen in table 5.

**Table 1:** Summary of screening result

	Number screened	Number positive		Number negative	
Males	97	13(13.40%)		84(86.59%)	
Females	91	14(15.38%)		77(84.61%)	

Total	188	27(14.36%)		161(85.64%)	

**Table 2:** Results of screening of males correlated with data obtained from the questionnaire

	**AGE GROUP**				
	18–27	28–37	38–47	48–57	58–67
No. Screened	44	24	17	10	2
Demographics/Socials					
Married	4	10	17	10	2
Single	40	14	-	-	-
Alcohol Consumption	21	14	10	4	2
Blood Transfusion	10	6	3	3	1
Tattoo/Body Piercing	6	5	4	3	1
Regular Exercise	26	10	9	4	1
HCV Positive	7	1	4	1	0
Percentage Positive	3.72	0.53	2.13	0.53	0
HCV Negative	37	23	13	9	2
Percentage Negative	19.68	12.23	6.91	4.79	1.06

**Table 3:** Results of screening of females correlated with data obtained from the questionnaire

	**AGE GROUP**				
	18–27	28–37	38–47	48–57	58–67
No. Screened	41	35	10	4	1
Demographics/Socials					
Married	10	27	10	4	1
Single	31	8	-	-	-
Alcohol Intake	2	7	3	1	-
Blood Transfusion	11	15	3	2	-
Tattoo/B. Piercing	35	31	8	4	1
Regular Exercise	21	20	2	-	-
HCV Positive	3	7	3	1	0
Percentage Positive	1.60	3.72	1.60	0.53	0
HCV Negative	38	28	7	3	1
Percentage Negative	20.21	14.89	3.72	1.60	0.53

**Table 4:** Analysis of the results in relation to data from questionnaire

VARIABLE	TOTAL NOS(%)	NOS OF POSITIVE (%)	P VALUE
**Marital status**			
Married	95 (50.53%)	19 (10.11%)	0.026
Single	93 (49.47%)	8 (4.26%)	
			
**Sex**			
Male	97 (51.60%)	14 (7.45%)	0.977
Female	91 (48.40%)	13 (6.91%)	
			
**Age**			
18–27	85 (45.21%)	10 (5.32%)	0.364
28–37	59 (31.38%)	8 (4.26%)	
38–47	27 (14.36%)	7 (3.72%)	
48–57	14 (7.45%)	2 (1.06%)	
58–67	3 (1.60%)	- (0.00%)	
			
**Blood transfusion**			
YES	54 (28.72%)	7 (3.72%)	0.728
NO	134 (71.28%)	20 (10.64%)	
			
**Tattoo/body piercing**			
YES	98 (52.13%)	12 (6.38%)	0.338
NO	90 (47.87%)	15 (7.98%)	
			
**Alcohol intake**			
YES	64 (34.04%)	7 (3.72%)	0.218
NO	124 (65.96%)	20 (10.64%)	
			
**Exercise**			
YES	92 (48.94%)	14 (7.45%)	0.743
NO	96 (51.06%)	13 (6.91%)	

**Table 5:** Results for liver enzyme tests

	Men		Women	
	AST (%)	ALT (%)	AST (%)	ALT (%)

Nos showing normal level	12(12.37)	10(10.31)	10(10.99)	9(9.89)
Nos showing abnormal level	2(2.06)	4(4.12)	3(3.29)	3(3.29)

## Discussion

A prevalence of 14.36% was established in the subjects sampled. When compared with reports by Halim and Ajayi [19] who recorded a prevalence of 5.8% – 12.3% in Nigeria and that reported by Udeani,*et al*. [20] who reported a prevalence of 13.6% in Jos, it is evident that the rate of transmission of HCV is on the increase. The highest seropositive values of 10(5.32%) and 8(4.26%) were found among subjects in the age bracket 18 – 27 years and 28 – 37 years. This is similar to values reported by Mcquillan *et al*. [21] with highest HCV prevalence being found among persons aged 20 – 49 years. The high seropositivity recorded in these groups may be as a result of their exposure to contaminated blood through blood transfusion. 21 of the 85 subjects in the age group 18 – 27 years had received blood transfusion. The subjects in this age group could also have been infected through the use of contaminated instrument during tattoo or body piercing [22]. We cannot rule out the fact that these subjects may develop chronic HCV leading to cirrhosis and subsequently DM. 41 of the 85 subjects in the age group 18 – 27 had tattoo or body piercing. Also 36 of the 59 subjects in the age group 28 – 37 years had tattoo or body piercing. Since this group constitute the workforce (productive age group), it could lead to loss of manpower especially when they suffer acute illness and are probably hospitalized. Males had a higher seropositivity of 7.45% than the females with 6.91% in agreement with the report by Alter [23] that males seem to be more predisposed to HCV than females. The higher seropositivity in male subjects of age group 18–27 could be due to the fact that they are more sexually active [21]. Although more females than males had received blood transfusions, had tattoos or body piercings, these may also have been sources of HCV infection in the males.

Amongst females, the highest seropositive value of 7(3.72%) was recorded in females in the age range 28 – 37 who were 35 in number. As much as 15 of them had received blood transfusion and 31 of them had had tattoo, ear or body piercing. Among the female subjects in the age group (28 – 37 years), 20 of them exercise frequently. Exercise is not directly a risk factor to HCV infection but lack of exercise (evidenced by a sedentary life style) could be a risk factor to Diabetes in high-risk (genetically predisposed) individuals. Such individuals are therefore advised to undertake more exercise as this improves glucose and lipid metabolism thus decreasing their risks of manifesting Diabetes as reported by Pan *et al*. [24]. Also most females in this age group (28 – 37 years) were married and could have been infected through sexual intercourse. Since most of the 'positives' feel within the age groups 18 – 27 and 28 – 37, it is evident that mostly younger people were infected, agreeing with the work of Kev and Francois [25].

Sixty four (64) subjects take alcohol, 14 of them testing positive for HCV. Alcohol intake is not directly a risk factor to HCV. However, it has been documented that there exists a synergy between alcohol and HCV. Increased alcohol consumption increases the risk of fibrosis leading to cirrhosis and consequently hepatocellular carcinoma [26] and probably ultimately to DM (in DM predisposed individuals). Alcoholic HCV infected patients have higher hepatic iron concentration than non alcoholic HCV positive patients. High iron concentration plays a role in liver damage and also increases the rate of HCV replication. Plasma levels of pro inflammatory cytokines like tumor necrosis factor- alpha are increased in acute alcoholic hepatitis and such cytokines induce insulin resistance and glucose intolerance which could consequently cause type II Diabetes mellitus [27].

Liver enzyme analysis on sero-positive subjects showed that liver damage is probable in most subjects with levels already above normal and some indicated early onset of liver disease. Subjects who were sero-positive to HCV infection could have contacted the infection through several modes of transmission and may not have been symptomatic in the acute stage of the infection. If the disease, however progresses to the chronic stage in future, aided by life style of alcohol consumption, it could lead to the development of extrahepatic complications including Diabetes mellitus. However in HCV seropositive individuals with family histories of Diabetes, there is evidence that life style intervention or pharmacologic agents (drugs) can reduce the development of Diabetes. Since current methods of treating Diabetes is inadequate, the most effective way to reduce the burden associated with Diabetes is to prevent Diabetes itself and this can be done by reducing the risks associated with the incidence of Diabetes.

## Conclusion

The findings in this study showed a significant prevalence of Hepatitis C virus infection among individuals with possible genetic predisposition to Diabetes mellitus. This may be on the increase due to the various sources and modes of transmission of the infection as identified in the study. It is also evident that certain risk factors, including seropositivity to HCV, a family history of Diabetes, obesity, sedentary life styles and malnutrition could lead to Diabetes mellitus. It is important therefore, for individuals who are genetically predisposed to be careful and avoid the risk of exposure to sources of HCV infection and undertake regular screening for HCV. Diabetes mellitus (DM) is a metabolic disease characterized by hyperglycemia. Mortality and morbidity from the disease makes it costly to both individuals and society in terms of quality of life and costs of care, hence the need for prompt diagnosis.

## Methods

### Study area and subjects

This study was carried out among individuals with family history of Diabetes living within and around Vom, Plateau State. A total of 188 subjects were screened (97 males and 91 females).

### Survey

A Questionnaire was administered to each subject prior to sample collection. This was done to obtain some demographic data, social behavior and possible risk factors engaged in by the subjects.

### Sample collection and processing

From each subject, 5 ml of peripheral blood was aseptically collected by venous puncture of the ante-cubital fossa region of the arm using a sterile needle and syringe. They were transferred into centrifuge tubes, allowed to retract and then centrifuged at 3000 rpm for 5 minutes to get clear supernatant sera. The sera were separated into clean and dry sample bottles, labeled and stored in a -20°C freezer prior to use.

### Detection of HCV antibodies

Serological screening for HCV antibodies was carried out using the Biotech third generation ELISA kit (HCV Ab Elisa 7/032A). The reagent employs both synthetic and recombinant HCV for the detection of antibodies to HCV in human serum. Positive samples were further analyzed spectrophotometrically for liver enzymes (aminotransferases, specifically ALT and AST) to ascertain the degree of liver damage.

### Statistical analysis

Data obtained were analyzed using the SPSS software.

## Competing interests

The authors declare that they have no competing interests.

## Authors' contributions

O.O.N: Conceived, coordinated and wrote up the findings of this research. J.A.N: Carried out the screening on the samples for HCV. G.O.N.E: Participated in the design of the study. A.O.O: Participated in the design of the study. Chika I. Nwosuh: Participated in the design of the study. E.M.O: Carried out the screening on the samples for HCV. L.A.O: Participated in sample collection. J.O.A: Collected samples and participated in the screening of samples for HCV. B.N.D: Carried out liver enzyme analysis on HCV sero-positive samples. I.O.N: Collected samples and participated in the screening of samples for HCV. G.O.A: Collected samples and participated in the screening of samples for HCV. A.A.C: Participated in the design and coordination of the research. All authors read and approved the final manuscript.
